# First reported case in an Irish flock of MCF- like systemic necrotizing vasculitis in sheep associated with ovine herpesvirus 2

**DOI:** 10.1186/s13620-024-00269-w

**Published:** 2024-05-04

**Authors:** Maresa Sheehan, Patricia A. Pesavento, Francis Campion, John Lynch, Shane McGettrick, Brian Toland, Aideen Kennedy

**Affiliations:** 1 Kilkenny Regional Veterinary Laboratory, Department of Agriculture, Food and the Marine, Kilkenny, Ireland; 2 Department of Pathology, Microbiology and Immunology, School of Veterinary Medicine, University of California, Davis, USA; 3 Teagasc, Animal & Grassland Research and Innovation Centre, Mellows Campus, Athenry, Co. Galway Ireland; 4 Archersfield Veterinary Clinic, Archersfield House, Kilkenny, Co. Kilkenny Ireland; 5 Sligo Regional Veterinary Laboratory, Department of Agriculture, Food and the Marine, Sligo, Ireland

**Keywords:** MCF- like systemic necrotizing vasculitis, Ovine gammaherpesvirus 2 (OvHV-2), Sheep, Ireland

## Abstract

**Background:**

Ovine gammaherpesvirus 2 (OvHV-2) is the causative agent of sheep associated malignant catarrhal fever (MCF). As sheep are the adapted host for OvHV-2, it is generally presumed that infection is not associated with disease in this species. However, a recent case review combined in-situ hybridisation, PCR and histopathology and correlated the viral distribution with systemic necrotizing vasculitis and concluded OvHV-2 was the likely agent responsible for sporadic, MCF-like vascular disease in sheep.

**Case presentation:**

Using similar methods this case study reports on the findings of the first reported cases in an Irish Flock of MCF- like systemic necrotizing vasculitis in sheep associated with OvHV-2. Sheep A, a 16-month-old Texel-cross hogget displayed signs of ill- thrift, Sheep B, a nine-month-old Belclare-cross lamb, was found dead having displayed no obvious symptoms. Both cases occurred on the same farm, however the animals were not related. Lymphohistiocytic vasculitis of various tissues was the predominant histopathological finding in both animals.

**Conclusion:**

By combining histopathology, PCR and in-situ hybridisation results, MCF- like systemic necrotizing vasculitis associated with OvHV-2 has been diagnosed for the first time in an Irish flock.

## Background

Malignant catarrhal fever (MCF) is a disease primarily of cattle caused by a group of MCF viruses (MCFV) from the genus *Macavirus*, a sub-family of Gammaherpesvirinae [1]. Among susceptible species, such as cattle, deer and bison, there is variability in the viral load, incubation period and the clinical signs reported [1]. Multiple tissues can be affected, including gastrointestinal, ocular, urinary, respiratory, skin and neurological tissues [1]. Clinical cases in cattle are considered to have a mortality rate approaching 100%, and recovery rarely occurs [2]. Histopathologically, regardless of the tissue affected, the hallmark of MCF is lymphocytic arteritis with necrosis of the tunica media [1].

As sheep are the adapted host for ovine gammaherpesvirus 2 (OvHV-2) [3], and because prevalence of infection is presumed to be high [4, 5], it is generally assumed that infection is not associated with disease in this species. Recently it has been proposed that infection with OvHV-2 can sporadically result in MCF in sheep, with proof of causality based on a combination of experimental disease, quantitative PCR [6], and in-situ hybridisation (ISH) [7]. A series of historic, sporadic cases of idiopathic systemic necrotizing arteritis in sheep have been confirmed to be MCF based on viral distribution that correlated well with necrotizing vasculitis [8]. Using similar methods this case study reports on the findings of two cases of MCF- like systemic necrotizing vasculitis in an Irish sheep flock.

## Case presentation

### Flock overview

The flock consisted of 15 purebred Belclare ewes, 25 cross breed ewes, three breeding rams (Belclare, Texel and Suffolk) and 75 lambs. The animals were mainly homebred, along with bought in rams (typically purchased annually). 18 lambs were purchased in the year before the first case (these were purchased from a known source and to the farmers knowledge this farm has not reported similar clinical signs to the current cases).

Purebred ewes are lambed in January and the remainder in March. The ewe to lamb weaning rate is 1.8–2.0 lambs per ewe mated. Lambs are weaned at 14 weeks of age and are sold to slaughter with the exception of ewe lambs kept for breeding and six pedigree ram lambs for sale as breeding stock. Grass is the primary diet with concentrate feeding occurring pre-lambing and to some finishing lambs. The flock is housed from November/December to March/April on silage/hay and concentrates.

Periodic soil sampling, forage analysis and mineral analysis has not to date identified any underlying imbalances. Lambs are drenched periodically with cobalt during the grazing season, but ewes rarely receive mineral supplementation bar magnesium lick buckets post-partum.

The health status is generally perceived as good by the farmer. There are occasionally cases of coccidiosis in lambs. Sheep are vaccinated for clostridial diseases and pasteurella pneumonia. There are occasional problems with orf but not to the extent where vaccination would be required. There are no other species on the farm although they occasionally use the same handling yard as another cattle herd.

Rare cases of unexplained ill thrift have been reported by the farmer.

### Case A

One of these cases of ill thrift was a 16-month-old Texel- cross hogget that was losing condition for 4 months and had signs of diarrhoea. This animal had been purchased as a lamb the year previously (none of the purchased cohorts have displayed similar signs). The hogget was unresponsive to treatment for fluke and worms. Faecal samples for McMaster examination and salmonella culture were considered negative. The animal was euthanised and presented to Kilkenny Regional Veterinary Laboratory for post-mortem examination. At the time of euthanasia this animal was housed indoors, on a grass silage and cereal based diet.

#### Post mortem Case A

Faecal staining was noted around the tail of the carcass. There were minimal gross findings; mild congestion in the lungs, the abomasal mucosa had a thickened appearance and the intestines were filled with watery green contents. *Escherichia coli* was cultured from the liver and lung. There were less than 50 strongyle eggs per gram on McMaster testing, no liver or rumen fluke eggs were detected, *Clostridium perfringens* ELISA tests were negative.

Negative PCR results were recorded for Maedi visna virus (MVV)*,* Caprine arthritis and encephalitis virus*, Mycoplasma ovipneumoniae, Mannheimia haemolytica, Pasturella multocida*, *Anaplasma phagocytophilum* and pestiviruses. The rumen pH was normal. Liver cobalt and copper levels were normal; kidney selenium was within normal range (Table 1).

**Table 1:** Tests performed and results of tests on both Case A and Case B

**Test**		**Case A**	**Case B**
Biochemistry (Tissue)	Kidney selenium Liver cobalt Liver copper	11.10 µmol/kg 01.55 µmol/kg 1.06 mmol/kg	Not tested 0.97umol/kg 0.73 mmol/kg
Biochemistry (Vitreous humor)	Beta-hydroxybutyrate Calcium Magnesium	Not tested Not tested Not tested	0.32 mmol/L 1.39 mmol/L 0.66 mmol/L
ELISA	*Clostridium perfringens*	Not detected	Not detected
Histopathology H&E	Abomasum Brain Intestine (& ZN stain) Kidney Liver Lung Turbinate (Case B only)	See histopathology findings	
Parasitology	McMaster Sedimentation: Liver & rumen fluke eggs	< 50 Strongyle epg Coccidia not detected Not detected	50 Strongyle epg Light Coccidia Not detected
PCR (Viruses)	Bluetongue *Caprine arthritis and encephalitis Virus* *Maedi Visna Virus* *Ovine gammaherpesvirus 2* Pestiviruses	Not detected Not detected Not detected Positive Not detected	Not detected Not detected Not detected Positive Not detected
PCR (Bacteria)	*Mycoplasma ovipneumoniae,* *Mannheimia haemolytica,* *Pasturella multocida,* *Anaplasma phagocytophilum* (tick borne fever)	Not detected Not detected Not detected Not detected	Not detected Not detected Not detected Not detected
pH	Rumen content	6.1	5.3
Routine culture	Intestine Lung Liver	E-coli E-coli E-coli	E-coli E-coli Sterile

Tissues were collected and fixed in 10% neutral-buffered formal saline for histologic examination: lung, liver, kidney, abomasum, intestine and brain were collected (sections of the turbinate were included in Case B). Tissues were routinely processed, embedded in paraffin, and 4-µm sections were stained with hematoxylin and eosin (H&E). Additional Ziehl Nielsen (ZN) stained sections of the intestines were also produced.

#### Histopathology

**Fig. 1:**
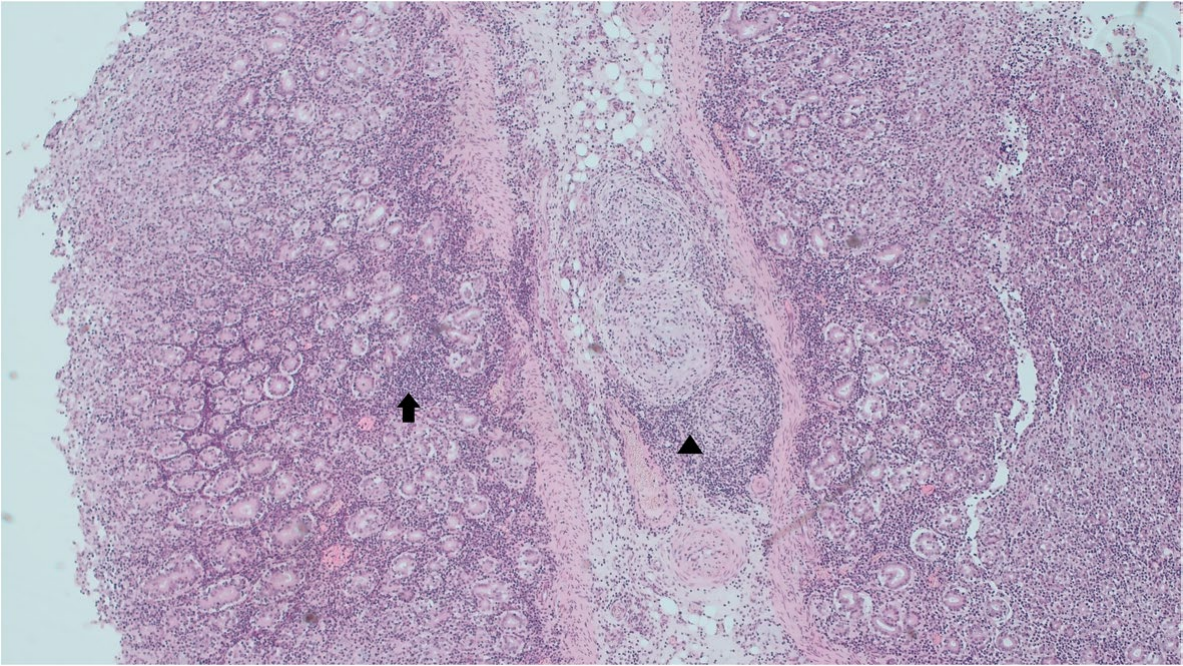
Case A- Lymphocytic enteritis (arrow) with perivascular accumulations of mainly mononuclear cells (arrowhead) and a vasculitis affecting primarily medium bore vessels (5 × H& E)

The main histopathology findings in Case A indicated a diffuse lymphocytic enteritis and systemic lymphohistiocytic vasculitis. There was a moderate to severe enteritis; the intestinal mucosa contained a dense infiltration of lymphocytes, and there was multifocal crypt abscessation. Multiple blood vessels in the submucosa appeared hypertrophic and were cuffed by a large number of lymphocytes and some macrophages (Fig. 1). ZN stains of the intestine were negative. There was diffuse mild interstitial pneumonia with mild BALT hyperplasia and mild angiocentric lymphocytic accumulations. There was a non-specific reactive hepatitis. A large number of lymphocytes and macrophages distended the portal areas and there was mild lipidosis. Multifocally some vessels in the meninges of the brain were hypertrophic with lymphocytic cuffing.

### Case B

Four months later, Animal B, a home bred nine-month-old Belclare-cross lamb, was found dead at pasture and was submitted to Kilkenny Regional Veterinary Laboratory for post-mortem examination. No symptoms were noted prior to death.

#### Post mortem Case B

On post-mortem examination the lamb showed signs of moderate dehydration. There was blood visible at the external nares. Cross section of the turbinates revealed inflamed and swollen turbinates with multifocal 1–2 mm nodules. There was undigested concentrates and some roughage in the rumen. There were loose contents in the small and large intestines.

*Escherichia coli* was cultured from the lung, low numbers of coccidial oocysts were detected, liver copper and cobalt were within normal ranges. Negative PCR results were recorded for Maedi visna virus, Caprine arthritis and encephalitis virus, *Mycoplasma ovipneumoniae, Mannheimia haemolytica, Pasturella multocida, Anaplasma phagocytophilum* and pestiviruses (Table 1). The rumen pH was marginally acidotic (pH 5.3).

#### Histopathology

**Fig. 2:**
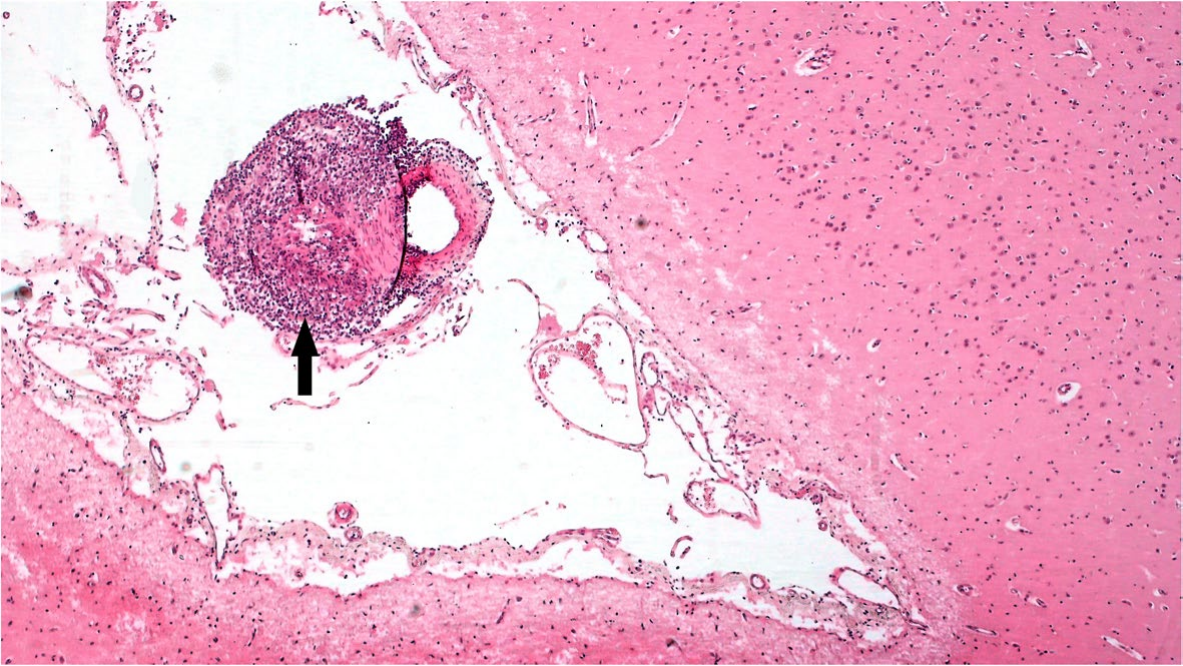
Case B- Meningoencephalitis with vasculitis. Arrow points to the lymphohistiocytic vasculitis (5 × H& E)

**Fig. 3:**
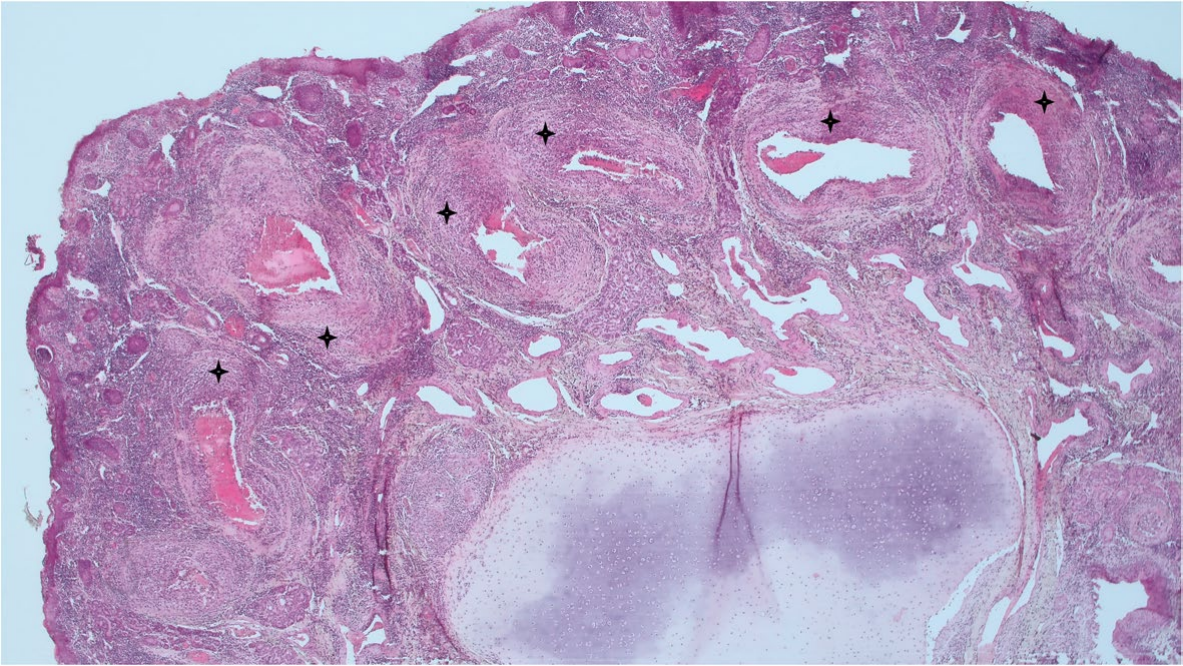
Case B- Nasal turbinates with multifocal to coalescing non-suppurative rhinitis with vasculitis characterised by marked lymphocytic and histiocytic infiltrations. Stars indicate some affected vessel walls (2.5 × H&E)

Histopathological examination of the brain revealed a multifocal meningoencephalitis with non-suppurative lymphohistiocytic vasculitis (Fig. 2), diffuse interstitial pneumonia with lymphohistiocytic vasculitis and perivascular lymphocytic cuffing, nephritis with vasculitis and non-specific reactive hepatitis with mild centrilobular necrosis. Examination of the turbinates showed a multifocal lymphohistiocytic vasculitis with large numbers of lymphocytes and macrophages in the connective tissue and surrounding the seromucous glands (Fig. 3).

**Fig. 4:**
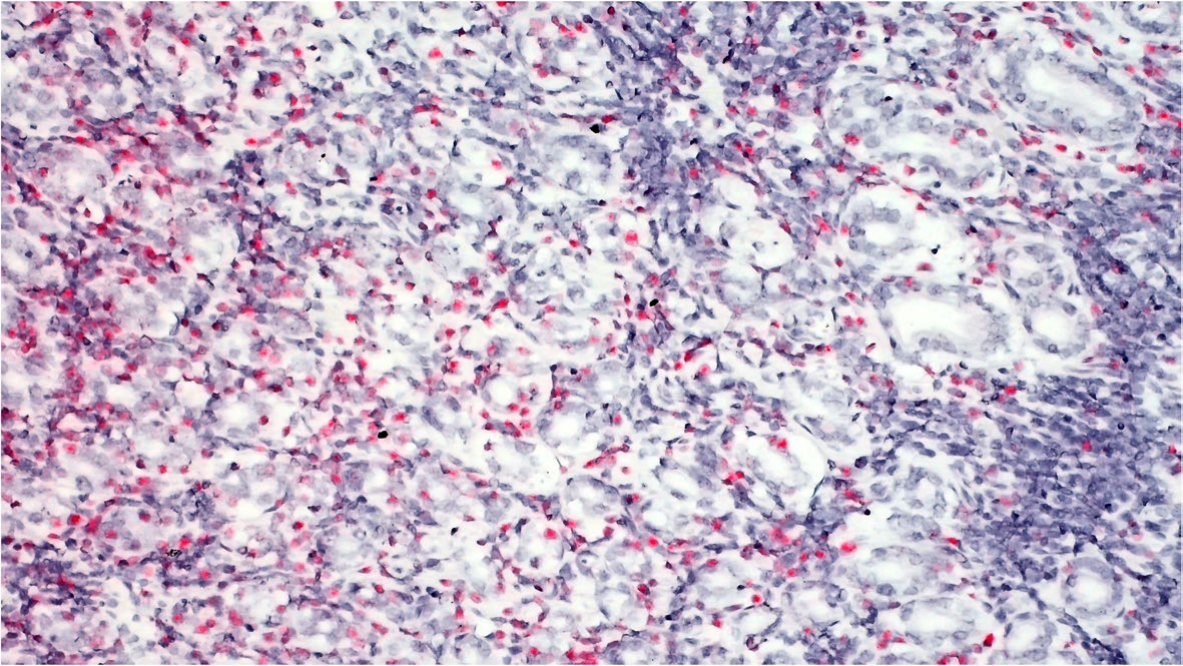
Case A- Chromogenic (red staining) In situ hybridization demonstrates OvHV-2 probe colocalization within mononuclear cells in intestines of sheep with lymphocytic enteritis (20x)

OvHV-2 PCR positive results were obtained in both cases with CT values less than 25. The OvHV-2 PCR assay was conducted in line with methods previously published by Cunha et al. [13] and Hüssy et al. [14]. Based on these findings formalin fixed paraffin embedded sections of brain and intestine were sent to Department of Pathology, Microbiology and Immunology, School of Veterinary Medicine, University of California, Davis, USA for further examination by ISH.

ISH was performed as described in Pesavento et al. (2019a). In brief, ISH was performed manually on 5-μm sections of formalin-fixed, paraffin-embedded tissue. Designed paired probe sets V-OvHv2-orf25-orf50 ACD (cat. 501,091) as 30ZZ targeting regions 49,813–50,552 of open reading frame 25 (ORF25) and 76,874–77,614 of ORF50 (excluding the intronic region) in OvHV-2 (GenBank #NC_007646) were used. Two negative controls were used to assess the specificity of the signal for OvHV-2. First, replicate sections of the slides in the study were tested with an unrelated, GC content-matched probe (diaminopimelate B (DapB)). The negative control in this case was an unrelated, DapB, bacterial probe.

**Fig. 5:**
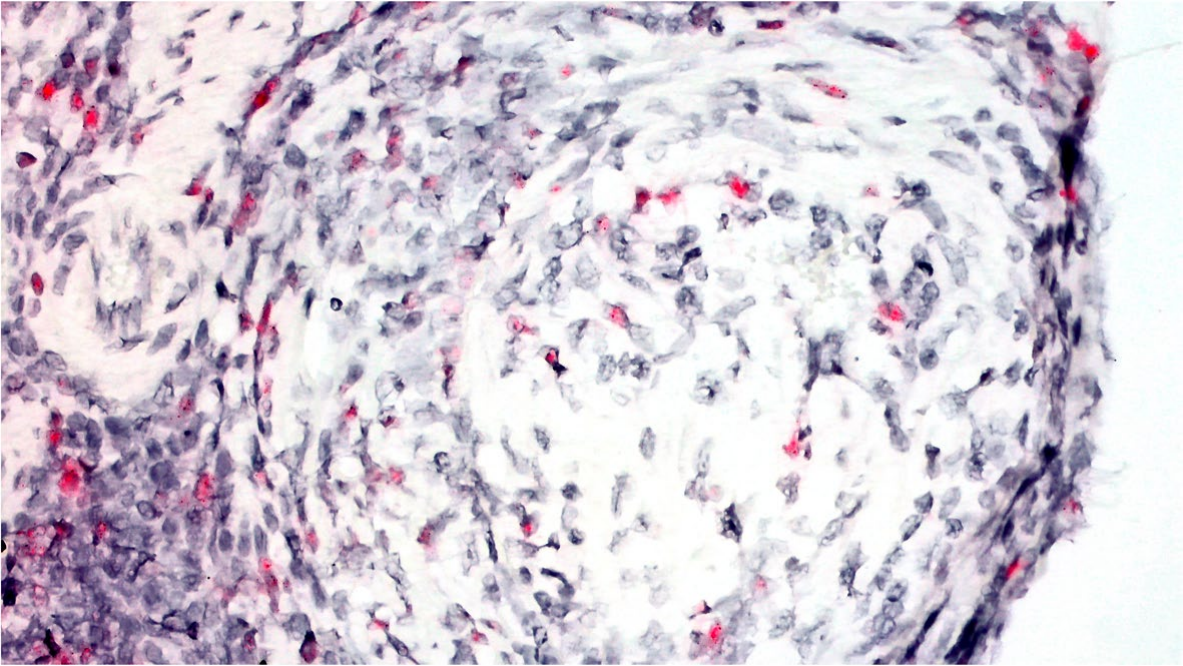
Case A- Chromogenic (red staining) In situ hybridization demonstrates OvHV-2 probe colocalization within mononuclear cells in intestines of sheep with lymphocytic enteritis (40x)

Intestinal tissues of case 1 were positive for OvHV-2 by ISH. The probe hybridization was detected in the nuclei of a subset of lymphocyte nuclei surrounding and penetrating the wall of some, but not all arteries. The affected arteries were muscular arteries of the submucosa. Probe hybridization was detected as well in abundant nuclei of lymphocytes within the lamina propria (Figs. 4 and 5).

In case B, ISH positive results were detected in the meningeal arteries of the brain. As decalcification destroys the hybridisation process, it was not possible to perform ISH on the nares.

Based on histopathology, PCR and ISH results it was concluded MCF was the most likely agent responsible for the lesions seen in both sheep.

## Discussion

In most sheep lifelong subclinical infection with OvHV-2 is established by 1 year of age. Li et al. (1998), reported most lambs to be born uninfected, with infection occurring after 2 months of age. Both sheep in this study were different ages at the time of death and it is not possible to infer when infection occurred. However, six- to nine-month-old lambs shed virus more frequently than adults [3], and it may suggest the cases were exposed to high levels of virus during adolescence. The cases reviewed in the Pesavento et al. (2019b) study ranged in age from 2.5 months to three years of age. Rae et al. (1994), reported a number of cases of lymphocytic enteritis and systemic lymphocytic vasculitis in sheep predominantly aged between five and twelve months. Ferreras et al. (2013), and Li et al. (2005), reported necrotising vasculitis in 9-month-old animals, with Ferreras et al. (2013), also reporting the condition in a 1–2-year-old; similar in age to Case A.

Wessels et al. (2017) previously described cases of systemic necrotising polyarteritis in multiple animals from the same flock. However, contrastingly, in the Wessels et al. (2017), report, two of the lambs had linked parentage (Beltex breed), whereas the cases in the current study were of unrelated parentage. Cases have previously been reported in Texel, Beltex, Assafs and cross breed animals [8–12]. Two of the lambs in Wessels et al. (2017), study were reported as having ill thrift and diarrhoea prior to death. Similar clinical signs were seen in Case A of this study. Although not confirmed as ‘MCF associated’ lymphocytic vasculitis, the cases in Rae et al. (1994) also reported weight loss and inappetence, with or without diarrhoea. Diarrhoea was also reported by Pesavento et al. (2019b). The clinical signs in the reported cases were variable and included lymphadenopathy [11, 12], respiratory symptoms [6, 12], ataxia [8], recumbency and weakness [10], swollen joints [10] and in one case hemoabdomen was noted [11]. Unusually the second case in this study displayed no clinical signs prior to death. While Case A was euthanized to investigate ill thrift, Case B was found dead, and it is likely the marked vascular changes in many organs were a contributory factor in the death. The involvement of the turbinates is unusual. While Li et al. (2005), reported rhinitis in experimentally induced infection, to the authors knowledge the lymphohistiocytic vasculitis of the turbinates has not previously been reported in naturally occurring infections in sheep.

Wessels et al. (2017), raised the possibility of an immune mediated component to the disease, similarly O’Toole et al. (2014), concluded that the pathogenesis of MCF involves a dysregulated immune response. Tick borne fever, known to cause immunosuppression was ruled out in the current cases. Additionally, no ‘stressful’ event could be identified as an initiating factor in disease manifestation. Important differentials for viral associated vasculitis in sheep were excluded including border disease and blue tongue. Flock investigations did not identify any agent that could contribute to an underlying immunosuppression, however the possibility of immunocompromise can’t be fully excluded. Additionally, the involvement of a co-microbial pathogen cannot be excluded as also noted by Pesavento et al. (2019b).

Systemic necrotising vasculitis associated with OvHV-2 is a condition rarely reported in sheep [8], and this study marks the first report in Ireland. As reported by others [8, 11, 12], PCR positive results for OvHV-2 were obtained. As many sheep are sub clinically infected with OvHV-2 [12] it is important to remember PCR positive results are insufficient evidence for diagnosis of MCF- like systemic necrotizing vasculitis in sheep. A combination of histopathology, ISH and PCR positive results is required for disease confirmation.

## Conclusion

By combining histopathology, PCR and ISH results MCF- like systemic necrotizing vasculitis associated with OvHV-2 has been diagnosed for the first time in an Irish flock. An unusual finding in one of the cases was lymphohistoicytic vasculitis of the turbinates. As decalcification destroys the hybridisation process, it was not possible to perform ISH on the nares to confirm OvHV-2 as the agent responsible for the turbinate lesions, however given the histopathology findings, examination of the turbinates in suspect cases is recommended. In spite of extensive negative test results the possibility of a comicrobial pathogen, or underlying immunosuppression can’t be excluded in these cases. In future cases with similar histopathological signs, it will remain important to exclude vasculitis inducing agents such as border disease and most notably blue tongue which currently is exotic to Ireland.

## Data Availability

Not applicable.

## Abbreviations

ISH: In Situ Hybridisation
OvHV-2: Ovine gammaherpesvirus 2
MCF: Malignant catarrhal fever
